# Systems biology—opportunities and challenges: the application of proteomics to study the cardiovascular extracellular matrix

**DOI:** 10.1093/cvr/cvw206

**Published:** 2016-09-15

**Authors:** Javier Barallobre-Barreiro, Marc Lynch, Xiaoke Yin, Manuel Mayr

**Affiliations:** King’s British Heart Foundation Centre, King’s College London, 125 Coldharbour Lane, London SE5 9NU, UK

**Keywords:** Cardiovascular diseases, Extracellular matrix, Post-translational modifications, Proteomics, Systems biology

## Abstract

Systems biology approaches including proteomics are becoming more widely used in cardiovascular research. In this review article, we focus on the application of proteomics to the cardiac extracellular matrix (ECM). ECM remodelling is a hallmark of many cardiovascular diseases. Proteomic techniques using mass spectrometry (MS) provide a platform for the comprehensive analysis of ECM proteins without a priori assumptions. Proteomics overcomes various constraints inherent to conventional antibody detection. On the other hand, studies that use whole tissue lysates for proteomic analysis mask the identification of the less abundant ECM constituents. In this review, we first discuss decellularization-based methods that enrich for ECM proteins in cardiac tissue, and how targeted MS allows for accurate protein quantification. The second part of the review will focus on post-translational modifications including hydroxylation and glycosylation and on the release of matrix fragments with biological activity (matrikines), all of which can be interrogated by proteomic techniques.

## 1. Introduction

Proteomic techniques using mass spectrometry (MS) provide a platform for the comprehensive analysis of proteins, thereby facilitating the implementation of systems biology approaches and circumventing the limitations of a traditional, reductionist approach adopted by techniques like western blotting that are based on a priori assumptions of the proteins to be investigated. Furthermore, proteomics is without the constraints of antibody-dependent protein detection and has the capability of detecting post-translational modifications (PTMs), which is beyond the means of gene expression platforms.^1^

Tissue fibrosis is a hallmark of most cases of cardiovascular disease (CVD) and includes modification and deposition of extracellular matrix (ECM). However, detailed studies on the cardiovascular ECM have been sparse due to the lack of analytical tools that facilitate comprehensive characterization of its components. In recent years, proteomics has been successfully applied to study the ECM, providing unprecedented insights into its biology and pathological remodelling.^2–5^ In the present review, we describe the utility of ECM proteomics as applied to cardiovascular research and the potential pitfalls. In addition, we highlight the means to overcome common proteomic challenges and present translational applications of proteomic datasets.

## 2. The ECM in cardiac disease

The ECM not only confers mechanical stability, but is also a reservoir for bioactive molecules. Remodelling of the ECM, including quantitative but also qualitative changes in composition, is a hallmark of CVD. Numerous studies have demonstrated that structural, but also non-structural ECM proteins play crucial roles during disease progression and normal cardiac physiology.

*Table1* summarizes important findings in clinical studies as well as in animal models of cardiac disease.^5–42^ Additional studies reported ECM proteins as potential biomarkers for cardiac pathologies^43^; these have been intentionally omitted from the table, the focus of which are ECM and ECM-associated proteins (i.e. extracellular proteases and non-structural proteins that bind to or regulate ECM) from a functional perspective. Most proteins included in the table were individually studied using antibodies and loss-of-function models in order to assign their relevance to disease. Proteomics can quantify most of these ECM proteins in a single experiment, leading to the identification of previously unreported links between ECM components in disease.^2–4^ For example, in a recent study we demonstrated that genetic deletion of biglycan was accompanied by an unexpected rise of aggrecan in murine aortas.^44^

**Table 1:** Role of ECM and ECM-associated proteins in cardiac disease

Protein	Clinical context	Main findings
Adiponectin	Cardiac remodelling (m)	Induces cell migration, MMP activation, and collagen remodelling via APPL1-AMPK signalling^6^
ADAMTS9	Developmental defects (m)	Haploinsufficiency leads to reduced versican cleavage, associated with cardiac anomalies^7^
Biglycan	MI (m)	Required for adaptive remodelling^8^
Cathepsin-K	AF (h, rb)	Increased levels and activity accompanied atrial changes linked to the AngII/ATR1R signalling pathway^9^
Cathepsin-S	MI (m)	Mediates fibroblast transdifferentiation during remodelling^10^
Collagen I	Dilated cardiomyopathy (m)	Point mutation induces cardiomyopathy^11^
Collagen VI	MI (m)	Absence improves cardiac function, structure, and remodelling^12^
Collagen XIV	Developmental defects (m)	Important for growth and structural integrity of the myocardium^13^
Collagen XV	Hypertension (m)	Necessary for remodelling. Deficiency predisposes to cardiomyopathy^14^
Connective tissue growth factor	Pressure overload (m)	Inhibition attenuates left ventricular remodelling and dysfunction^15^
Decorin	Left ventricular assist device implantation (h)	Ameliorates adverse remodelling by mediating TGF-beta inhibition^16^
	MI (m)	Absence leads to abnormal scar tissue formation^17^
Fibronectin	MI (m, h)	Essential for progenitor cell response during cardiac repair^18^
	MI (m)	Lack of EDA domain promotes survival and prevents adverse remodelling^19^
Fibulin-2	MI (m)	Loss protects against progressive ventricular dysfunction^20^
Laminin alpha-4	Dilated cardiomyopathy (h, z)	Mutations cause human cardiomyopathy via defects in cardiomyocytes and endothelial cells^21^
Lumican	Hypertrophy (m)	Deficiency results in cardiomyocyte hypertrophy with altered collagen assembly^22^
Mimecan	MI (m, h)	Prevents cardiac dilatation and dysfunction via collagen strengthening^23^
MMP-14	Pressure overload (m)	Mediates pro-fibrotic signalling, leading to alterations in interstitial fibrosis and diastolic function^24^
MMP-28	MI (m)	Deletion exacerbates cardiac dysfunction and rupture by inhibiting M2 macrophage activation^25^
TIMP-2	Pressure overload (m)	Loss leads to exacerbated left ventricular dysfunction and adverse ECM remodeling^26^
MMP-9	AF (p, h)	Increased gelatinase activity contributes to atrial ECM remodelling^27^^,^^28^
	MI (h,m)	Crucial for generation of bioactive collagen I fragments that promote scar formation after MI^5^
	MI (m)	Deletion leads to decreased collagen accumulation and left ventricular enlargement^29^
MMP-2	MI (m, r)	Contributes to ischemia-reperfusion injury, and deletion/inhibition prevents cardiac rupture^30^^,^^31^
Osteopontin	MI (m)	Deletion leads to left ventricular dilatation and reduced collagen deposition after MI^32^
Periostin	MI (r)	Blockade of Exon 17 preserves cardiac performance^33^
	Pressure overload (m)	Deletion results in less fibrosis and hypertrophy^34^
Perlecan	Developmental defects (m)	Perlecan is critical for heart stability^35^
SPARC	MI (m)	Mediates early ECM remodeling^36^
Tenascin-C	Pressure overload (m)	Accelerates fibrosis by activating macrophages via the integrin αVβ3/nuclear factor-κB/interleukin-6 axis^37^
	MI (m)	May aggravate left ventricular remodelling and function^38^
Thrombospondin-1	Pressure overload (m)	Protects myocardium by modulating fibroblast phenotype and ECM metabolism^39^
	MI (d, m)	Role in preventing expansion of healing myocardial infarcts^40^
Thrombospondin-4	Pressure overload (m)	Regulates myocardial fibrosis and remodelling^41^
Versican	Developmental defects (m)	Associated with chamber specification, septation, and valvulogenesis in the developing heart^42^

MMP, matrix metalloproteinase; ADAMTS, a disintegrin and metalloproteinase with thrombospondin domains; TIMP, tissue inhibitor of metalloproteinases; SPARC, secreted protein acidic and rich in cysteine; MI, myocardial infarction; AF, atrial fibrillation; m, mouse; h, human; rb, rabbit; z, zebrafish; r, rat; p, pig; d, dog.

## 3. ‘In antibodies we trust’

Until recently, the identification of proteins in a tissue or a protein lysate has been limited by the availability of antibodies that recognise certain regions (epitopes) of a protein of interest. Antibodies have been and continue to be an important component of the armamentarium for protein research but they are not without limitations. While antibody arrays overcome the restriction to only one protein, ECM proteins tend to be under-represented in arrays. The main issues of antibody-based protein quantification, however, remain the same: (i) Usually only a small portion of the protein (epitope) is recognised by an antibody. Protein detection by antibodies relies on the presence of unmodified epitopes. In ECM proteins, however, common PTMs include hydroxylation, glycosylation or fragmentation. Due to epitope masking, ECM proteins may not be detectable by antibodies. (ii) Antibodies are often not commercially developed to target proteins in species beyond the commonly used mice and rats such as canine and porcine models for myocardial infarction,^2^^,^^45^^,^^46^ rabbit and goat models for studies involving atrial fibrillation^9^^,^^47–49^ and sheep as models of dilated cardiomyopathy.^50^ While some anti-human or anti-mouse antibodies will cross-react, many others will not recognize their target protein in different species or will display a high degree of non-specific binding. Vice versa, proteins in the bovine serum supplements of cell cultures can be detected not only in the conditioned media but also in the cell lysates.^51^

**Figure 1:**
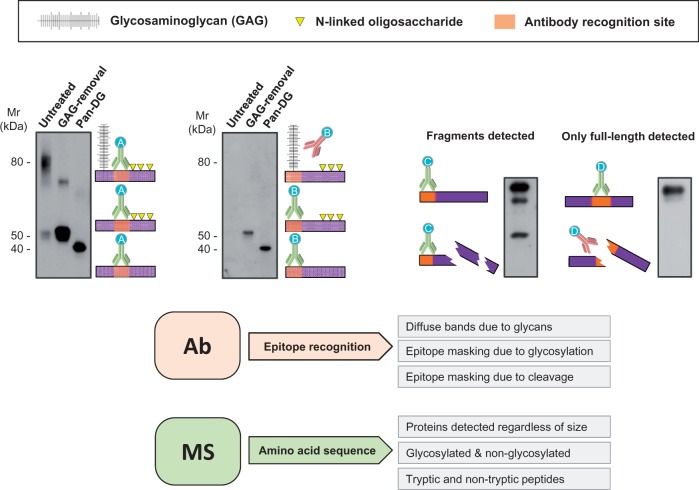
Antibody limitations. Detection by antibodies relies on binding to specific regions (epitopes) of the target protein. PTMs such as glycosylation or fragmentation may hinder epitope accessibility. Antibody A recognises non-glycosylated regions and always yields detection independently of sugar removal (left panel). Antibody B recognises epitopes in the vicinity of glycosylated regions. Therefore, recognition is only achieved after deglycosylation. Similarly, if protein fragmentation occurs, only antibody C, which recognises an intact portion, reveals a degradation pattern. Antibody D targets a region affected by fragmentation and can only detect the intact epitope. Consequently, information about degradation is missed. Proteomics interrogates peptides across the whole sequence and allows for consideration of variable modifications at the amino acid level. Different protein forms can therefore be identified and quantified. GAG, glycosaminoglycan; Pan-DG, pan-deglycosylation.

**Figure 2:**
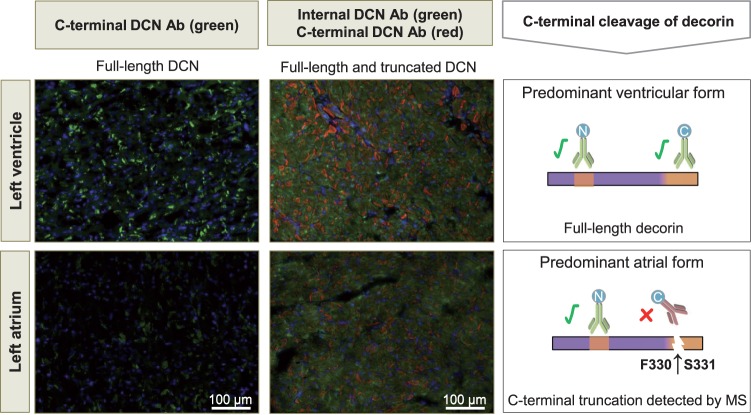
MS to explain discrepancies between different antibodies. An antibody against a C-terminal epitope (green on left panels, red on right panels) results in less intense staining for decorin (DCN) in the atrium compared to the left ventricle. An antibody against a different epitope (green on right panel) shows no such difference in staining intensities. This may be explained by cleavage of decorin at the C-terminus, which was detected in the atrium using MS.

Contrary to antibodies, proteomics does not rely on recognition of one specific epitope and can be applied across species. Moreover, the use of MS allows for determination of changes that occur at the protein level (i.e. amino acid modifications) (*Figure 1*). For example, we demonstrated using MS that the C-terminus of decorin, a small leucine-rich proteoglycan, is often cleaved in the left atrium but not in the ventricle.^52^ MS data provided an explanation why the use of different antibodies for the same target protein yielded very different results (*Figure 2*).

## 4. ECM revisited by proteomics

Proteomics is the study of the complete protein component of a living organism, tissue or cell and yields unbiased data without a priori knowledge. The workhorse of modern proteomics is the mass spectrometer and although it is not a new technology *per se*, it was for a long time confined to areas outside the biological sciences. However, it was the advent of matrix-assisted laser desorption ionization (MALDI)^53^ and in particular of electrospray ionization (ESI)^54^—which enables liquid chromatography (LC) systems to be interfaced directly to mass spectrometers—that MS branched from analytical chemistry into biology.

The gold standard for contemporary proteomics is LC-tandem MS (LC-MS/MS). Briefly, the LC column separates the peptides (typically generated by digesting proteins with trypsin) in the analyte prior to ionisation and subsequent MS analysis. In addition to recording the mass of the peptide ions, MS/MS technologies induce the subsequent fragmentation of these precursor ions. The masses of these fragment ions can therefore be used to delineate the amino acid sequence of the peptide. The availability of annotated protein sequence databases and algorithms that match the observed MS/MS spectra to protein entries have been crucial for the biomedical application of MS to study proteins.^55^^,^^56^ MS data can also be aligned to databases generated using DNA or RNA sequences to infer amino acid sequences. Current MS technologies now allow for the characterization of the ECM composition and turnover in CVD in unprecedented detail that is not possible using other techniques.^2–5^^,^^52^^,^^57–59^

## 5. Extraction of ECM proteins

Over the past years our group has focused on ECM remodelling in cardiac^2^^,^^52^^,^^60^^,^^61^ and vascular tissues^3^^,^^4^^,^^57^^,^^59^ using proteomics. In a previous review, we highlighted the potential of proteomics when applied to systems biology.^62^ A recent review by Chang *et al*.^63^ has focused on clinical applications of ECM proteomics (i.e. biomarker discovery and tissue engineering). In this review, we discuss how MS can be used to assess ECM composition in CVD.

Contemporary mass spectrometers have exceptional sensitivity, providing detection at attomole concentrations.^64^ However, such sensitivity is largely confined to pure solutions and not yet achievable in complex biological samples. For example, the dynamic range of proteins present in plasma spans 10 to 11 orders of magnitude (e.g. 4 × 10^10^ pg/ml for albumin compared to few pg/ml for some interleukins).^65^ Current MS instrumentation can only resolve 4–6 orders of magnitude. While proteomics offers a comprehensive analysis of high abundant proteins it has not yet overcome the difficulties of analysing low abundant proteins in complex samples. Unlike PCR or antibody-based techniques, proteomics lacks the ability to amplify low abundant proteins to aid detection and instead relies on enriching the target proteome.

For instance, cardiac ECM proteins are markedly less abundant than cytosolic and mitochondrial proteins.^46^ Thus, studies that use whole tissue lysates for proteomic analysis inevitably mask the identification of the less abundant ECM constituents. With cardiac tissue this is exacerbated due to the higher cellular content.^2^ Accordingly, methods that enrich for ECM proteins have received considerable interest of late and principally focus on removing plasma contaminants and soluble cellular proteins.^57^^,^^66^

**Figure 3:**
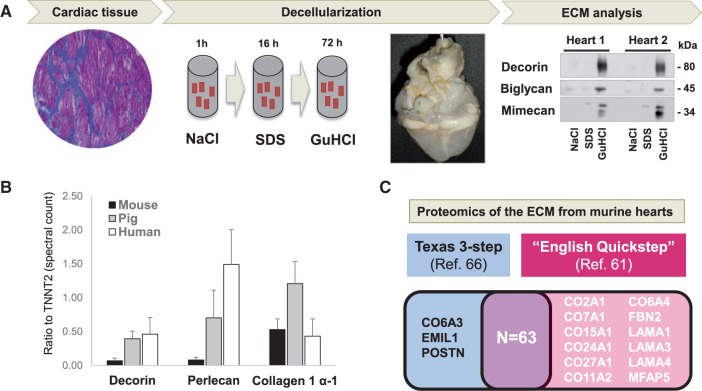
Enrichment of cardiac ECM proteins. (*A*) Our previously published 3-step ECM extraction method for cardiac tissue is based on decellularization and ensures enrichment and detection of ECM proteins**.** The image shows a decellularized heart after prolonged SDS perfusion. The ECM is solubilized by GuHCl and analysed using proteomics. (*B*) Smaller species display higher levels of cardiac cellularity as measured by the ratio of 3 members of different ECM protein classes and the cardiac-specific troponin T (TNNT2, y-axis). (*C*) Proteins identified in murine hearts using the Texas 3-step^66^ extraction method compared to those identified by our previously published method (see Drozdov *et al.*^61^)*.* Most proteins are identified by both methods. Unlike the Texas 3-step method, our ‘English Quickstep’ method did not include an analysis of the remaining pellet after GuHCl extraction.

While the inherent insolubility of many ECM proteins lends itself to effective enrichment by decellularization, subsequent proteomic analysis requires all proteins to be solubilized. Standard lysis buffers are not effective for ECM solubilisation. Instead, we implemented a stepwise extraction of vascular ECM proteins.^57^ This involves treating vascular tissues with sodium chloride (NaCl) to remove plasma proteins and extract loosely bound extracellular proteins before decellularizing the tissue with sodium dodecyl sulfate (SDS). Each incubation step takes 4 h. Solubilisation of mature ECM proteins is finally achieved by treatment with guanidine hydrochloride (GuHCl) which destabilizes the ionic, disulfide-dependent protein conformations in large aggregating proteoglycans (versican, aggrecan, etc.), small proteoglycans (decorin, biglycan, etc.), cell-attachment glycoproteins such as type VI collagen, fibronectins, laminins, and basement membrane components.^67^ The method was later adapted for the use in porcine cardiac tissue by reducing the incubation time for NaCl and prolonging the SDS treatment^2^ (*Figure 3A*). In smaller animal models (i.e. mouse, rat) cardiac cellularity is proportionally higher compared to that of larger animal models such as pig or goat (*Figure 3B*). With increased cellularity, decellularization is more difficult to achieve and may require additional enrichment steps, i.e. for glycoproteins or glycopeptides.^52^

Others have adopted similar workflows to extract ECM proteins in a number of tissues.^66^^,^^68–70^ Of note is the Texas 3-step extraction method by Lindsey’s group.^66^ In their method, applied to mouse hearts, a similar sequential extraction consisting of NaCl and GuHCl extraction steps as well as the SDS decellularization^2^^,^^4^^,^^57^ are performed. In addition, the Texas 3-step method includes further extraction of the insoluble protein pellet after incubation in GuHCl for 48 h. Notably, the vast majority of ECM proteins are identified in the GuHCl fraction. The pellet, however, contains few polymerized proteins, which are not extracted by our ‘English Quickstep’ method (*Figure 3C*).

In a recent proteomics study, Johnson *et al*.^71^ studied the human cardiac ECM from cadaveric donor hearts. Decellularization was achieved after perfusion with high SDS concentration (i.e. 10 times greater than that used in our protocol) for more than 3 days. This yields a simplified ECM, but the ECM proteins will be denatured and ECM-associated proteins will be lost during prolonged incubation with such a high concentration of detergents. The study of ECM using MS approaches described below, requires a gentler extraction method from snap-frozen tissues that strikes a balance between removal of cellular components while preserving ECM-associated proteins.

## 6. Discovery proteomics

Discovery proteomics refers to the use of proteomics as a hypothesis-free tool to globally profile the proteome of a given sample. In discovery proteomics, bottom-up or shotgun proteomics is based on the analysis of peptides generated after enzymatic digestion of a protein mixture. Digested peptides are separated by LC before MS/MS analysis. In comparison to gene expression analysis ECM proteomics offers certain advantages. First, many diseases manifest over years. Therefore, although transcript levels provide a window into cellular activity at the time of harvest, they merely provide an indirect assessment of protein synthesis at a single time point. When studying dynamic entities such as the ECM, transcript levels become more extraneous, particularly as nascent ECM proteins are incorporated into the existing matrix, and actual ECM protein abundance is determined by the balance of protein synthesis, deposition, and degradation.

There are multiple MS approaches that can be applied to yield accurate quantitation. However, each approach comes with distinct trade-offs.^62^ Label-free methods can provide relative quantification in simple mixtures. In complex mixtures, isotopic labelling should be employed, which allows multiplexing of samples. For instance, stable isotope labelling with amino acids in cell culture (SILAC) is based on metabolic labelling of proteins *in vitro* with amino acids containing heavy (e.g. ^13^C) stable isotopes. Fully labelled SILAC mice have also been generated.^72^ Methods for protein labelling are based on the use of isobaric tags, such as isobaric tags for relative and absolute quantitation (iTRAQ) or tandem mass tag (TMT).^73^ Isobaric tags have the same chemical structure but different isotope substitutions. When samples are labelled with different tags, they can be subsequently mixed in equal portions, and the protein abundance from the different samples can be assessed by comparing the abundance of peptides labelled with the different tags in a single LC-MS/MS run. Although these tagging methods overcome issues such as technical reproducibility of LC-MS/MS runs, labelling is only introduced after protein digestion and therefore, unlike SILAC, isobaric tags do not allow for *in vivo* or *in vitro* labelling but have been used for quantitative comparisons using tissue samples.^74^^,^^75^

## 7. Targeted proteomics

**Figure 4:**
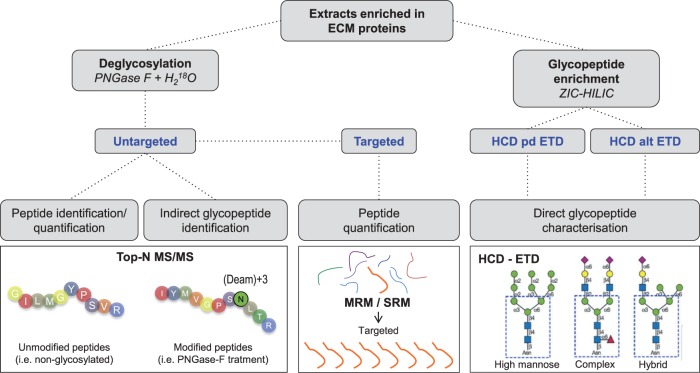
MS strategies for ECM characterisation. Untargeted proteomics is appropriate for discovery experiments where no a priori information is available. When a delimited number of targets of interest are known a priori, targeted proteomics offers a robust method for detection and quantification. Novel MS methods such as a combination of higher energy collision dissociation (HCD) and electron transfer dissociation (ETD) allow for characterisation of complex PTMs including glycosylation. ZIC-HILIC, zwitterionic hydrophilic interaction LC; Pd, product-dependent; Alt, alternating.

The discovery proteomics approach is largely limited by the scan speed as peptides are selected for fragmentation based on abundance. This stochastic process results in a bias towards the more abundant proteins.^62^ In contrast to discovery proteomics, targeted proteomics focuses on a predetermined group of proteins of interest (e.g. ECM proteins). Proteotypic peptides unique to these proteins are quantified in what is known as selected reaction monitoring (SRM) or multiple reaction monitoring (MRM).^76^ The targeted approach increases selectivity, sensitivity, and accuracy and enables simultaneous measurement of hundreds of transitions in a single LC-MS/MS run.^76^ The transitions for proteotypic peptides will be interrogated as a surrogate of total protein levels, but peptides not included in the search (e.g. non-annotated PTMs) are not detected.^55^ This approach is particularly useful to detect CVD biomarkers, as Domanski *et al*.^77^ demonstrated in a study that also included ECM biomarkers of fibrosis. Moreover, targeted proteomics constitutes a robust method to validate findings obtained from discovery experiments (*Figure 4*).^3^

## 8. Post-translational modifications

ECM proteins are often modified by PTMs,^1^ most notably hydroxylation and glycosylation.

### 8.1. Collagen hydroxylation

Collagens are the major fibril-forming proteins in the ECM and they consist of a basic triple-helical conformation. The triple helix increases molecular stability and provides resistance to tensile stress. Although many types of collagen exist, a consistent pattern can be observed for amino acid sequences of all collagens; each chain contains enriched triplet repeats consisting of the sequence Xaa-Pro-Gly, where Xaa is any amino acid, Pro is proline, and Gly is glycine. Prolines within these domains become hydroxylated under the action of prolyl-hydroxylases.^78^ Hydroxyprolines provide the substrate for the formation of hydrogen bonds between the adjacent collagen alpha chains. Prolyl-4-hydroxylases and prolyl-3-hydroxylase catalyse the hydroxylation of specific proline residues. The former enzyme reacts on proline with the minimum sequence Xaa-Pro-Gly and the latter appears to require a Pro-4Hyp-Gly (Hyp is hydroxyproline) sequence.^78^^,^^79^ Hydroxylation is a stable, non-reversible PTM that adds +15.99 Da (i.e. an oxygen atom) to proline. Xaa-Pro-Gly domains are rare in ECM proteins other than collagens, and this confers specificity to the acquisition of this PTM.

Similar to prolyl-hydroxylases, lysyl-hydroxylases catalyse the hydroxylation of lysine, which is critical for collagen stability. The specifics of lysine hydroxylation are beyond the scope of this review, and are discussed elsewhere.^80^ Adding hydroxylation as a variable modification, improves identification and quantification of collagen levels in disease.^3^^,^^68^ Ascorbic acid (vitamin C) is a key cofactor for prolyl-4-hydroxylase, and its deficiency causes defects in collagen assembly.^81^ Inhibition of this enzyme has been shown to affect left ventricular remodelling after myocardial infarction in rats.^82^ In this study only proline:hydroxyproline ratios were assessed. MS provides assessment of hydroxylation with concomitant assignment to specific collagen types.

### 8.2. Glycosylation

Glycosylation is an enzymatic process through which a glycan is covalently attached to a second biomolecule. Glycosylation is a very common form of PTM of ECM proteins. Attached glycans affect ECM protein structure and function by influencing its folding, solubility, aggregation, and/or degradation behaviour.^83^ Indeed, aberrant glycoforms are already approved as biomarkers for cancer.^84^ In cardiac tissue, Montpetit *et al*.^85^ showed that aberrant glycosylation of extracellular domains alters ion channel activity.

There are two main glycosylation types in mammals: N-glycosylation occurs at the carboxamido nitrogen on asparagine residues (Asn) of secreted/membrane proteins within the consensus sequence Asn-Xaa-Thr/Ser, where Xaa is any amino acid except for proline.^86^ The second main type of glycosylation is O-glycosylation, in which sugar residues attach to serine and threonine residues (Ser, Thr) or, to a much lesser extent to hydroxyproline and hydroxylysine.^87^ The latter two are particularly abundant in collagens and add an additional level of regulation to collagen biosynthesis. If both present, O-glycosylation occurs after N-glycosylation. Moreover, O-glycosylation is not restricted to secreted proteins and to date, no consensus sequences have been identified for this PTM.^88^ ECM proteins may be extensively modified by addition of N- and O-linked large and repetitive glycosaminoglycans (GAGs) and shorter and diverse N- and O-linked oligosaccharides. Aberrant glycosylation can lead to pathological abnormalities and disease. In the last decade, proteomics has emerged as a powerful platform to characterize glycosylation profiles of ECM proteins, including the cardiovascular field.^52^^,^^58^^,^^89^ There are two major strategies that can be used to study glycoproteins by MS (*Figure 4*).

#### 8.2.1 Indirect MS methods

A common large-scale strategy utilizes a glycopeptide or glycoprotein enrichment step followed by glycan removal. Glycopeptides are usually enriched using lectins, hydrophilic interaction LC, hydrazide or graphite. The method of choice will determine the type of glycopeptides that will ultimately be enriched.^90^^,^^91^ After enrichment, PNGase-F is used to enzymatically remove the glycan moiety from asparagine residues, serving two purposes: Firstly, the core peptide can be analysed without interference from sugars during MS/MS and secondly, PNGase-F via a deamidation reaction converts the asparagine to aspartic acid. This conversion is characterized by a 0.984 Da mass shift that can be detected using MS. Moreover, if the reaction is performed in the presence of $H_{2}^{18}$O, it instead leads to a 2.99 Da mass shift, indicative for the presence of glycosylation at that position. Using this methodology in rat hearts, Parker *et al.*^58^ identified 1556 N-linked glycosites from 972 protein groups. This study provided information on the changes in glycosylation following ischemia and reperfusion. Enzymatic deglycosylation allows for the separate analysis of the core protein and glycan,^92^ but the link between the glycans and peptides is lost.

#### 8.2.2 Direct MS methods

The combined analysis of the glycan motif (glycomics) and the protein (proteomics) forms the field of glycoproteomics. For such analysis, proteins in the sample are first digested into peptides, followed by glycopeptide enrichment using zwitterionic hydrophilic interaction LC (ZIC-HILIC)^93^ or alternative approaches.^91^^,^^94^ Recently, the combination of higher energy collision dissociation (HCD) and electron transfer dissociation (ETD) have facilitated direct MS analysis of glycopeptides. HCD fragmentation breaks glycosidic bonds, whereas ETD preserves the glycan attachment and fragments the peptide backbone, providing peptide sequence information.^89^ Direct analysis of intact glycopeptides has rarely been applied in the cardiovascular context. Our study by Yin *et al*.^89^ characterised the glycopeptides of secretomes from human endothelial cells. More recently, we have characterized the glycosylation profile of human cardiac ECM proteins.^52^

### 8.3. Reversible PTMs on ECM proteins

Glycosylation and hydroxylation are among the most common PTMs in ECM proteins. Importantly, they constitute non-reversible modifications, but reversible PTMs such as phosphorylation and sulfonation also occur. For example, the transmembrane collagen XVII can be phosphorylated and this mechanism regulates shedding of its ectodomain.^95^ Similarly, phosphorylation of osteopontin inhibits vascular calcification.^96^ In a study by Lundby *et al*.^97^ proteomics was used to identify phosphosites on 14 different rat tissues including hearts. Phosphopeptides were enriched using titanium dioxide. Notably, many previously unrecognized phosphosites were reported in ECM proteins including several collagens and non-collagenous ECM proteins such as laminins, fibronectin, versican, and decorin to name a few. This methodology was effective on fresh animal tissues, but has yet to be applied to the context of cardiac disease. Challenges to its application will include preservation of short-lived PTMs in patient samples and during sample preparation.

## 9. Fragmentation of ECM proteins

Proteolytic fragmentation of ECM proteins by secreted proteases controls their localisation, activation, and interaction, adding an additional layer of regulation for tissue processes. Using experimental data, databases/algorithms such as MEROPS and PROSPER have been created to calculate probability matrices for target protease sequences.^98–100^ This is a valuable resource for research and is particularly useful when used in conjunction with proteomics. Standard proteomics pipelines work with digested protein mixtures (i.e. trypsin digestion) to screen for the abundance of proteins in a tissue. Trypsin cleaves C-terminally to lysine (Lys, K) or arginine (Arg, R) residues. However, proteases other than trypsin can be present in samples and the endogenous proteolytic activity may give rise to non-tryptic peptides. In a study from Stegemann *et al*.^59^, we described a number of potential proteolytic targets for various MMPs in the vasculature after addition of these proteases to human vascular tissue explants.

When searching for protease targets, appropriate controls are needed (i.e. healthy or non-digested tissues) in order to avoid reporting artefactual cleavages that may arise from experimental processing, or identifying those that are part of normal physiological turnover. Moreover, the addition of broad-spectrum protease inhibitors during extraction reduces the chance of producing artefactual fragmentation. More sophisticated methods include free C- or N-terminal labelling of endogenous protease-generated fragments prior to digestion for MS analysis.^101^ For example, the TAILS proteomics approach (isotope-based N-terminal labelling) has been successfully applied by Prudova *et al.*^102^ to analyse the degradome of MMP-2 and MMP-9. The same authors used a similar methodology to characterize the degradation of proteolytic fragments in human platelets.^103^ Ultimately, after identification of cleavage sites, targeted proteomics can be used to study the abundance of ECM fragments in clinical samples.

**Figure 5:**
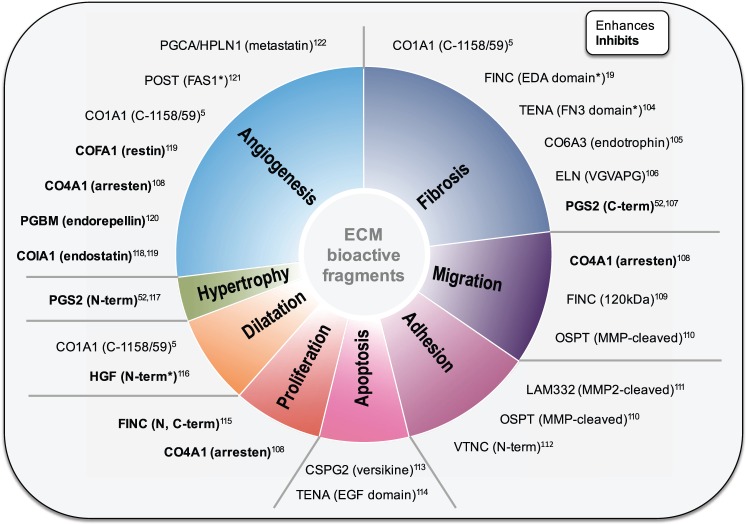
Biological activity of ECM fragments. Fragments derived from a variety of ECM proteins (i.e. matrikines) exert functions that regulate diverse cellular and tissue processes. Proteomics offers a tool for the analysis of known ECM fragments as well as the discovery of previously unknown fragments with functions potentially important for cardiac physiology and putative therapeutic targets. *Indicates putative fragments with activities only characterised after exogenous administration. CO1A1, collagen alpha-1(I) chain; FINC, fibronectin; EDA, extra domain A; TENA, tenascin; FN3, fibronectin type III domain; CO6A3, collagen alpha-3(VI) chain; ELN, elastin; PGS2, decorin; CO4A1, collagen alpha-1(IV) chain; OSTP, osteopontin; LAM332, laminin 332; VTNC, vitronectin; CSPG2, versican; EGF, epidermal growth factor-like domain; HGF, hepatocyte growth factor; COIA1, collagen alpha-1(XVIII) chain; PGBM, perlecan; COFA1, collagen alpha-1(XV) chain; POST, periostin; FAS1, fasciclin-like domain; PGCA, aggrecan; HPLN1, hyaluronan and proteoglycan link protein 1.

Specific biological activities have been attributed to certain ECM proteolytic fragments (*Figure 5*).^5^^,^^19^^,^^52^^,^^104–122^ The term matrikines has been proposed for these fragments. This should not be confused with the term matricryptins, which is more accurately applied to ECM protein domains that are unexposed (and therefore inactive) unless the protein is subject to fragmentation-derived conformational changes. For example, C-terminal cleavage of collagens XV and XVIII, generates restin and endostatin, respectively. Both fragments exert anti-angiogenic activity *in viv*o.^119^ Other collagen types also generate biologically active fragments, e.g. collagens IV and VI, which are highly expressed in the cardiac ECM^2^, as reviewed elsewhere.^123^ The large proteoglycan versican is cleaved by proteases from the matrix metalloproteinase (MMP) and a disintegrin and metalloproteinase with thrombospondin motifs (ADAMTS) families.^124^ Versikine is generated by N-terminal cleavage of versican by ADAMTS-1/4, and influences cell proliferation and apoptosis locally.^113^ Endorepellin, a C-terminal peptide from perlecan exerts anti-angiogenic effects.^120^ Last, non-structural ECM proteins also release fragments, e.g. the small leucine-rich proteoglycan decorin releases decorunt and other fragments, that exert local regulatory roles over cytokines and growth factors.^52^^,^^107^^,^^117^ Recently, we demonstrated that decorin is fragmented in the cardiac ECM. We detected C- and N-terminal non-tryptic cleavage sites on decorin by MS. The resulting cleavage products may regulate growth factor availability.^52^ Using similar approaches, the Lindsey group identified cleavage products derived from collagen I that promote scar formation after MI.^5^

## 10. Conclusions

The application of MS constitutes one of the biggest technological advances introduced to protein research. It offers an unbiased platform to analyse global protein expression and holds potential in facilitating novel insights. As recently highlighted in a scientific statement of the American Heart Association^55^; it is anticipated that proteomics research will further our understanding of mechanisms of CVD with one important aspect being the elucidation of ECM composition in healthy and diseased cardiovascular tissues. To achieve this goal, bioinformatics approaches should be applied for interpreting the protein datasets and extract the biologically relevant information. Undoubtedly, the amount of data generated by proteomics represent an analytical challenge. In this regard, special attention should be paid to ECM fragments as they hold potential for two purposes: from a diagnostic perspective, they leak from tissues and when released into the blood stream can be used as biomarkers for CVD. Secondly, since many ECM fragments are biologically active, they not only hold potential as therapeutic targets but also as modifiable therapeutic agents—to date an underexplored avenue of cardiovascular medicine.
